# New Insights Into the Treatment of Hyperlipidemia: Pharmacological Updates and Emerging Treatments

**DOI:** 10.7759/cureus.63078

**Published:** 2024-06-24

**Authors:** Seema Abbasi, Adnan Khan, Muhammad W Choudhry

**Affiliations:** 1 Internal Medicine, Sutter Health, Stockton, USA; 2 Cardiology, St. Joseph’s Medical Center, Stockton, USA; 3 Interventional Cardiology, St. Joseph’s Medical Center, Stockton, USA

**Keywords:** evinacumab, inclisiran, bempedoic acid, lomitapide, proprotein convertase subtilisin/kexin type 9 (pcsk9) inhibitors, ezetimibe, fibrates, statins, familial hypercholesterolemia, hyperlipidemia

## Abstract

Cardiovascular diseases are the leading causes of global mortality and morbidity. Hyperlipidemia is a significant risk factor for atherosclerosis and subsequent cardiovascular diseases. Hyperlipidemia is characterized by imbalances in blood cholesterol levels, particularly elevated low-density lipoprotein cholesterol and triglycerides, and is influenced by genetic and environmental factors. Current management consists of lifestyle modifications and pharmacological interventions most commonly consisting of statins. This review paper explores pathophysiology, management strategies, and pharmacotherapies including commonly used well-established medications including statins, fibrates, and ezetimibe, exciting novel therapies including proprotein convertase subtilisin/kexin type 9 (PCSK9) inhibitors, and RNA interference therapies (inclisiran), lomitapide, and bempedoic acid, highlighting their mechanisms of action, clinical efficacy, and safety profiles. Additionally, emerging therapies under clinical trials including ApoC-III inhibitors, DGAT2 inhibitors, ACAT2 Inhibitors, and LPL gene therapies are examined for their potential to improve lipid homeostasis and cardiovascular outcomes. The evolving landscape of hyperlipidemia management underscores the importance of continued research into both established therapies and promising new candidates, offering hope for more effective treatment strategies in the future.

## Introduction and background

Cardiovascular diseases are among the leading causes contributing to higher rates of morbidity and death in both developed and developing nations. Hyperlipidemia is one of the main risk factors for atherosclerosis and the ensuing cardiovascular disorders [1,2]. Hyperlipidemia is known as an imbalance in blood cholesterol levels characterized by elevated low-density lipoprotein cholesterol and lower levels of high-density lipoprotein cholesterol (HDL-C). Types of hyperlipidemias include pure hypercholesterolemia, mixed hyperlipidemia, which is characterized by increased levels of both triglycerides and cholesterol, and hypertriglyceridemia [3]. Hyperlipidemia is a significant risk factor for atherosclerotic cardiovascular disease (ASCVD), which can increase the risk of cardiovascular events, including coronary artery disease, myocardial infarction, stroke, and peripheral arterial disease. Elevated HDL-C levels (≥60 mg/dL) can help lower the risk of ASCVD because HDL-C helps the body eliminate cholesterol [3,4]. Hyperlipidemia is thought to be the cause of 29.7 million DALYS (disability-adjusted life years) or 2% of all DALYS and 2.6 million deaths (4.5% of total deaths) [5].

## Review

Pathophysiology of hyperlipidemia

Multiple genetic and environmental factors are linked to dysregulation of lipid metabolism, including increased synthesis, impaired clearance, or both, contributing to the development of hyperlipidemia. Modifiable risk factors include dietary habits characterized by high consumption of trans fats or saturated fatty acids, smoking, sedentary lifestyle, and obesity [6]. Other secondary reasons for increased levels of low-density lipoprotein-cholestrol (LDL-C) include type 2 diabetes mellitus, chronic renal disease, hypertension, biliary obstruction, and hypothyroidism [6]. Specific drugs, such as glucocorticoids, cyclosporine, and diuretics, can also lead to an elevation in LDL-C levels [1].

Genetics can contribute to the development of hyperlipidemia, making understanding its genetic underpinnings crucial for diagnosis, management, and treatment. Different genetic conditions including loss of function, gain of function, and genetic deletions lead to different forms of hypercholesterolemia. Familial hypercholesterolemia (FH) is an autosomal dominant genetic condition caused by mutations in low-density lipoprotein receptor (LDLR) gene resulting in defective or absent LDLR leading to high levels of LDL. Mutations in the apolipoprotein B (APOB) gene impair the ability of LDL to bind to its receptor. Gain of function in the proprotein convertase subtilisin/kexin type 9 (PCSK9) results in increased degradation of LDL receptors. These changes result in decreased LDL metabolism and reduced binding of LDL particles to the LDLR, raising LDL cholesterol levels [7-10].

Familial combined hyperlipidemia is a polygenic disorder characterized by elevated levels of triglycerides, LDL cholesterol, and often reduced HDL cholesterol. Common genes implicated include USF1, APOA1, APOB, and APOE. Familial hypertriglyceridemia is often polygenic, but mono-genes like LPL, APOC2, APOA5, GPIHBP1, and LMF1 genes can be implicated in development in its development.

LDLR mutation is the most common form of genetic mutation resulting in FH [11,12]. Patients with FH are at a higher risk of developing coronary heart disease compared to those without FH [12]. FH can be classified into two types: homozygous familial hypercholesterolemia (HoFH) and heterozygous FH (HeFH). HeFH is more prevalent in the United States, impacting roughly 1 in 500 people, and is linked to LDL-C levels ranging from 200 to 450 mg/dL [13]. Individuals diagnosed with heterozygous FH frequently experience early onset of cardiovascular disease, typically in the 40s dash 50s if untreated [10]. HoFH is a less common condition, involving around 1 in 300,000 to 1,000,000 individuals, but is characterized by significantly greater levels of LDL-C compared to HeFH (450 to >1000 mg/dL), associated with a very early onset of cardiovascular disease in childhood or adolescence. Untreated individuals with HoFH may experience mortality prior to reaching 20 years of age [13-15].

An analysis of the National Health and Nutrition Examination Survey (NHANES) found that the prevalence of FH was comparable among males and females; it varied among different ethnic groups. Mexican Americans had the lowest prevalence of FH, while whites, blacks, and other Hispanics had the highest prevalence [13].

Management

Management of hyperlipidemia involves a combination of non-pharmacological measures/lifestyle modifications, and pharmacotherapy consists of medication commands sometimes advanced therapies to reduce lipid levels and to minimize ASCVD. The utilization of clinical tools, such as ASCVD risk estimator by the American College of Cardiology/American Heart Association (ACC/AHA), can be advantageous in assessing the risk of individual patients. This risk-calculating tool has the potential to forecast cardiovascular events including coronary events and stroke in individuals aged 40 to 79, belonging to non-Hispanic white and African American populations, regardless of gender. However, it is important for physicians to be aware of the limitations associated with these risk predictors.

Non-pharmacologic management

Lifestyle interventions play a crucial role in the management of hyperlipidemia. Restricting saturated fat consumption and engaging in regular aerobic exercise are effective strategies for managing moderate hyperlipidemia, leading to notable enhancements in the lipid profile.

Dietary modification

Diet alone may not be effective in normalizing plasma cholesterol levels since only a small portion of blood cholesterol, 15%-20% comes from the food we eat. However, it plays a significant supplementary role to medical therapy and reduces the required dosage of medications. Dietary medications are more successful in patients with hypertriglyceridemia. In fact, lifestyle modifications including dietary modification and weight loss management are all needed to manage patients with mild to moderate hypertriglyceridemia [16]. Common dietary interventions for hyperlipidemia include decreasing overall food consumption and reducing portion, reducing saturated fat and cholesterol with monounsaturated and polysaturated fats by limiting intake of red meat, choosing lean meat like fish and poultry, and avoiding full-fat dairy products and processed food. Incorporating certain foods with favorable effects on the lipid profile, such as plant sterols and soluble fiber, can lead to a reduction of LDL-C levels by 6%-14%. Additionally, it is advisable to eliminate alcohol in patients with hypertriglyceridemia [17,18]. Achieving and maintaining a healthy weight through a combination of diet and exercise improves the lipid profile. For individuals with severe hyperlipidemia, it can be advantageous to seek the assistance of a certified dietician [19].

Exercise

Thirty to 60 minutes of moderately intensive exercise is recommended three to five times per week. Exercise not only helps maintain or reduce calorie intake, preventing weight gain and fighting obesity, but it also enhances insulin sensitivity, leading to improved breakdown of fats and promotion of the breakdown of lipoproteins high in triglycerides, and thus improves lipid profile [16]. Considering the absence of effective pharmacologic treatments specifically designed for individuals with low HDL-C, the primary approach to managing these patients is to focus on lifestyle adjustments that aim to reduce the risk of ASCVD. These factors encompass engaging in consistent physical activity, achieving an optimal body weight, quitting smoking, and adhering to a nutritious diet, all of which contribute to an increase in HDL-C levels. Undoubtedly, it is crucial for patients with dyslipidemia to manage additional ASCVD risk factors, such as quitting smoking and controlling high blood pressure and blood glucose levels [16].

Pharmacotherapy

Pharmacological interventions are often required, especially in individuals with persistent appointment inspired lifestyle modifications are those are higher risk of ASCVD. The most common pharmacotherapy for hyperlipidemia consists of statins and ezetimibe. Bile acid sequestrants (BAS) are less frequently used. PCSK9 inhibitors are often used as monotherapy or in combination with statins to achieve optimal lipid control. Besides PCSK9 inhibitors, emerging therapies consist of antisense oligonucleotides targeting apolipoprotein B (apoB) synthesis, and monoclonal antibodies against angiopoietin-like 3 (ANGPTL3) and apolipoprotein(a) [apo(a)].

Statins

Statins are the cornerstone of pharmacotherapy for hypercholesterolemia. Statins work by inhibiting 3-hydroxy-3-methylglutaryl-coenzyme A (HMG-CoA) reductase. This action reduces synthesis of cholesterol within hepatocytes, which then triggers a compensatory response by upregulation of LDL receptors on liver cells. These receptors bind to LDL particles in blood and facilitate increased LDL clearance. Statins also reduce production of very low-density lipoprotein (VLDL) in the liver, which is a precursor to LDL. Statins can cause 30% to 50% decrease in LDL levels. This reduction depends on the specific drug, dosage, pharmacogenetic variables, and the patient's compliance [20-22]. As a result, this decreases the harmful effects that LDL has on the arterial wall. Meta-analysis of 27 randomized statin trials revealed a 9% and 21% reduction in all-cause mortality and atherosclerotic cardiovascular disease events respectively for every 1 mmol/L (38.7 mg/dL) decrease in LDL-C [23-25]. Approximately 10% of individuals experience bothersome myalgias, which may decrease adherence to statin. This common side effect is reversible and pose no hazard to health [25-28]. Higher statin doses can slightly raise the risk of acquiring diabetes in people who are already predisposed to the condition and would likely have developed it regardless [25]. There are seven FDA-approved statins including lovastatin, simvastatin, pravastatin, fluvastatin, atorvastatin, rosuvastatin, and pitavastatin (Table 1).

**Table 1:** Major statin trials AMI: Acute myocardial infarction; CHD: coronary heart disease; CV: cardiovascular; CI: confidence interval; HR: hazard ratio; LDL: low-density lipoprotein; AFCAPS/TexCAPS: Air Force/Texas Coronary Atherosclerosis Prevention Study; MIRACL: Myocardial Ischemia Reduction with Acute Cholesterol Lowering; HPS: Heart Protection Study; PROSPER: Pravastatin in elderly individuals at risk of vascular disease; ALLHAT-LLT: The Antihypertensive and Lipid-Lowering Treatment to Prevent Heart Attack Trial; ASCOT-LLA: Anglo-Scandinavian Cardiac Outcomes Trial—Lipid Lowering Arm; CARDS: Collaborative Atorvastatin Diabetes Study; PROVE IT-TIMI 22 Pravastatin or Atorvastatin Evaluation and Infection Therapy–Thrombolysis in Myocardial Infarction 22; ASPEN: Atorvastatin Study for Prevention of Coronary Heart Disease Endpoints in Non-Insulin-Dependent Diabetes Mellitus; JUPITER: Justification for the Use of Statins in Prevention: an Intervention Trial Evaluating Rosuvastatin

Study Name/ Publication Year	Sample Size	Inclusion Criteria	Study Type (Primary/Secondary Prevention)	Statin Type, Dose and comparison	Median Follow-Up	LDL Reduction	Primary End Point	Secondary End Point	HR and 95% CI
WOSCOPS (1995) [29]	6,595	Men aged 45-64 with moderate hypercholesterolemia and no prior MI	Primary Prevention	Pravastatin 40 mg vs. placebo	4.9 years	26% reduction	31% reduction in CHD events: 248 events in the statin group vs. 174 events in the placebo group	22% reduction in all-cause mortality: 148 deaths in the statin group vs. 113 deaths in the placebo group	HR: 0.69 (95% CI: 0.57-0.83)
AFCAPS/TexCAPS (1998) [30]	6,605	Men and women with average cholesterol and no prior heart disease	Primary Prevention	Lovastatin 20-40 mg vs. placebo	5.2 years	25% reduction	37% reduction in first major coronary events: 116 events in the statin group vs. 183 events in the placebo group	Not specified	HR: 0.63 (95% CI: 0.50-0.79)
MIRACL (2001) [31]	3,086	Patients with acute coronary syndrome	Secondary Prevention	Atorvastatin 80 mg vs. placebo	16 weeks	40% reduction	No significant difference in death, non-fatal MI and cardiac arrest. 16% reduction in recurrent ischemic events: 208 events in the statin group vs. 256 events in the placebo group	Not specified	HR: 0.84 (95% CI: 0.70-1.00)
HPS (2002) [32]	20,536	Adults aged 40-80 with coronary disease or other occlusive arterial disease, diabetes, or treated hypertension	Secondary Prevention	Simvastatin 40 mg vs. placebo	5 years	25% reduction	24% reduction in major vascular events: 1,986 events in the statin group vs. 2,425 events in the placebo group	18% reduction in coronary death rate. 13% reduction in all-cause mortality: 1,328 deaths in the statin group vs. 1,507 deaths in the placebo group	HR: 0.76 (95% CI: 0.72-0.81)
PROSPER (2002) [33]	5,804	Men and women aged 70-82 with risk of or existing vascular disease	Primary and Secondary Prevention	Pravastatin 40 mg vs. placebo	3.2 years	34% reduction	15% reduction in coronary events: 408 events in the statin group vs. 473 events in the placebo group	No significant reduction in all-cause mortality: 252 deaths in the statin group vs. 259 deaths in the placebo group	HR: 0.85 (95% CI: 0.74-0.97)
ALLHAT-LLT (2002) [34]	10,355	Hypertensive patients with moderate hypercholesterolemia	Primary Prevention	Pravastatin 40 mg vs. usual care	4.8 years	17% reduction	No significant reduction in all-cause mortality: 818 deaths in the statin group vs. 835 deaths in the usual care group	No significant reduction in CHD events: 142 deaths in the statin group vs. 148 deaths in the usual care group	HR: 0.99 (95% CI: 0.89-1.11)
ASCOT-LLA (2003) [35]	19,342	Hypertensive patients with at least three other cardiovascular risk factors	Primary Prevention	Atorvastatin 10 mg vs. placebo	3.3 years	35% reduction	36% reduction in coronary events: 100 events in the statin group vs. 154 events in the placebo group	27% reduction in stroke incidence: 89 strokes in the statin group vs. 121 strokes in the placebo group	HR: 0.64 (95% CI: 0.50-0.83)
CARDS (2004) [36]	2,838	Patients with type 2 diabetes and no prior history of cardiovascular disease	Primary Prevention	Atorvastatin 10 mg vs. placebo	3.9 years	40% reduction	37% reduction in major cardiovascular events (acute coronary heart disease events, coronary revascularisation, or stroke): 83 events in the statin group vs. 127 events in the placebo group	48% reduction in stroke incidence: 21 strokes in the statin group vs. 39 strokes in the placebo group	HR: 0.63 (95% CI: 0.48-0.83)
PROVE IT-TIMI 22 (2004) [37]	4,162	Patients with acute coronary syndrome	Secondary Prevention	Atorvastatin 80 mg vs. Pravastatin 40 mg	2 years	51% reduction with Atorvastatin	16% reduction in primary end points: 343 events in the Atorvastatin group vs. 430 events in the Pravastatin group	Not specified	HR: 0.78 (95% CI: 0.69-0.89)
A to Z (2004) [38]	4,497	Patients with acute coronary syndrome	Secondary Prevention	Simvastatin 40 mg vs. 80 mg	2 years	28% reduction with high dose	No significant reduction in primary end points (CV death, non-fatal MI, readmission for AMI and stroke): 343 events in the high-dose group vs. 361 events in the low-dose group	Not specified	HR: 0.89 (95% CI: 0.77-1.03)
MEGA (2006) [39]	7,832	Japanese patients with hypercholesterolemia	Primary Prevention	Pravastatin 10-20 mg vs. diet therapy alone	5.3 years	18% reduction	33% reduction in coronary heart disease events: 66 events in the statin group vs. 97 events in the diet group	Not specified	HR: 0.67 (95% CI: 0.49-0.91)
ASPEN (2006) [40]	2,410	Patients with type 2 diabetes and history of cardiovascular disease	Secondary Prevention	Atorvastatin 10 mg vs. placebo	4 years	29% reduction	No significant reduction in primary cardiovascular end points	Not specified	HR: 0.89 (95% CI: 0.73-1.08)
JUPITER (2008) [41]	17,802	Patients with elevated high-sensitivity C-reactive protein (hsCRP) and low to normal LDL levels	Primary Prevention	Rosuvastatin 20 mg vs. placebo	1.9 years	50% reduction	44% reduction in major cardiovascular events: 142 events in the statin group vs. 251 events in the placebo group	20% reduction in all-cause mortality: 198 deaths in the statin group vs. 247 deaths in the placebo group	HR: 0.56 (95% CI: 0.46-0.69)
HOPE-3 (2016) [42]	12,705	Patients at intermediate risk of cardiovascular disease without established CV disease.	Primary Prevention	Rosuvastatin 10 mg vs. placebo	5.6 years	26.5% reduction	24% reduction in cardiovascular events: 235 events in the statin group vs. 304 events in the placebo group	Not specified	HR: 0.76 (95% CI: 0.64-0.91)

Fibrates

Fibrates are effective in managing hyperlipidemia, particularly hypertriglyceridemia, by activating a nuclear receptor, PPAR-alpha, and modulating the expression of genes involved in lipid metabolism. Fibrates enhance expression and activity of lipoprotein lipase (LPL), an enzyme that hydrolyzes triglycerides in VLDL and chylomicrons. These medications also reduce the expression of ApoC-III, an inhibitor of LPL; this further promotes the catabolism of triglyceride-rich particles [43,44]. They significantly reduce triglycerides, modestly increase HDL-C, and can provide additional cardiovascular benefits [45,46]. A systematic review and statistical analysis of 13 randomized controlled trials with a total of 16,112 participants demonstrated that fibrates have a beneficial effect in comparison to placebo in preventing the combined occurrence of stroke, myocardial infarction (MI), and cardiovascular death [47]. The FIELD and ACCORD studies did not yield any substantial evidence supporting the use of fibrates in cardiovascular prevention. The FIELD trial provided valuable insights into the potential benefits of fenofibrate therapy in this patient population. While fenofibrate did not significantly reduce the primary endpoint of coronary events, it demonstrated significant reductions in secondary endpoints, including non-fatal myocardial infarction and coronary revascularization procedures [48]. The ACCORD trial did not find a significant reduction in the risk of major cardiovascular events with the addition of fenofibrate to simvastatin therapy [49]. However, neither of the trials expressly required participants to have a baseline triglyceride (TG) level at the beginning of the study. A later subgroup analysis revealed that persons with hypertriglyceridemia saw a significant reduction in cardiovascular risk compared to those with lower triglyceride levels. The role of pemafibrate as an additional medication in reducing the residual risk of ASCVD in patients with high levels of TGs was investigated in the PROMINENT trial. However, the study was halted prematurely due to lack of effectiveness [50].

Overall, these studies suggest that there is not enough evidence that fibrates prevent significant cardiovascular events in patients with mild to moderate TG with a level between 150 and 500 mg/dl. Fenofibrate and gemfibrozil are commonly used fibrates. Bezafibrate is not available in US, and ciprofibrate is used in some countries. Common side effects are similar to statins consistent of myalgia, myopathy, and elevated liver enzymes necessitating periodic monitoring of liver function tests (Table 2).

**Table 2:** Major fibrate trials CHD: Coronary heart disease; HR: hazard ratio; CI: confidence interval; LDL: low-density lipoprotein; TG: triglyceride; HHS: Helsinki Heart Study; VA-HIT: Veterans Affairs Cooperative Studies Program High-Density Lipoprotein Cholesterol Intervention Trial; BIP: Bezafibrate Infarction Prevention; FIELD: Fenofibrate Intervention and Event Lowering in Diabetes; PROMINENT: Pemafibrate to Reduce Cardiovascular Outcomes by Reducing Triglycerides in Patients with Diabetes

Study Name/ Publication Year	Sample Size	Inclusion Criteria	Study Type (Primary/Secondary Prevention)	Fibrate Type, Dose and comparison	Median Follow Up	LDL Reduction	TG Reduction	Primary End Point	Secondary End Point	HR and 95% CI
HHS (1987) [51]	4,081	Men aged 40-59 with primary hypercholesterolemia	Primary Prevention	Gemfibrozil 1,200 mg/day vs. placebo	5 years	10% reduction	35% reduction	34% reduction in CHD death or nonfatal MI	Reduction in total mortality not statistically significant	HR: 0.66 (95% CI: 0.47-0.93)
VA-HIT (1999) [52]	2,531	Men with coronary artery disease and low HDL-C (<40 mg/dL)	Secondary Prevention	Gemfibrozil 1,200 mg/day vs. placebo	5.1 years	No significant change in LDL	31% reduction	22% reduction in CHD death or nonfatal MI	No significant difference in total mortality	HR: 0.78 (95% CI: 0.65-0.94)
BIP (2000) [53]	3,090	Patients with coronary heart disease and low HDL-C (<45 mg/dL)	Secondary Prevention	Bezafibrate 400 mg/day vs. placebo	6.2 years	No significant change in LDL	21% reduction	No significant reduction in primary endpoint of fatal and nonfatal MI	No significant difference in total mortality	HR: 0.92 (95% CI: 0.79-1.08)
FIELD (2005) [54]	9,795	Patients with type 2 diabetes	Primary and Secondary Prevention	Fenofibrate vs. placebo	5 years	No significant change in LDL	29% reduction	No significant reduction in primary endpoint of coronary events	Significant reduction in total cardiovascular events	HR: 0.89 (95% CI: 0.75-1.05)
PROMINENT (2022) [55]	10,497	Patients with type 2 diabetes and elevated triglycerides	Secondary Prevention	Pemafibrate vs. placebo	5 years	27% reduction	31% reduction	No significant reduction in primary endpoint of major cardiovascular events	Not specified	HR: 0.88 (95% CI: 0.78-1.00)

Ezetimibe

Ezetimibe is highly tolerable with negligible adverse effects. Ezetimibe targets and inhibits Niemann-Pick C1-like 1 (NPC1L1) protein, which is found on the brush border of enterocytes in the small intestine. NPC1L1 is responsible for uptake of cholesterol for our intestinal lumen into enterocytes. By inhibiting NPC1L1, ezetimibe reduces the absorption of dietary cholesterol, and cholesterol secreted in bile [56]. The usual 10 mg daily dose of ezetimibe effectively results in 18% to 25% reduction in LDL-C levels. The Improve-IT Trial enrolled 18,144 patients diagnosed with ACS, showed that by incorporating ezetimibe into statin medication, it was possible to decrease LDL-C levels from 1.8 to 1.4 mmol/L over a period of seven years. This reduction in LDL-C was linked to an additional ~7% decrease in major adverse cardiovascular events. Notably, this effect was particularly prominent in patients with diabetes [57]. Ezetimibe is recommended as a secondary treatment option in clinical practice guidelines [2,58,59]. It is usually prescribed as an adjuvant therapy to statins to achieve the target LDL goals and as monotherapy in patients who cannot tolerate statins.

Bile acid sequestrants

BAS are a valuable class of lipid-lowering agents using in combination with other agents, or monotherapy in patients with statin intolerance. BAS can reduce LDL-C levels by approximately 15-30% when used as monotherapy. BAS are hydrophilic resins with a positive charge that bind to negatively charged bile acids in the intestine, creating insoluble complexes that are eliminated in the stool. Consequently, there is a decrease in the reabsorption of bile acids, leading to an increased hepatic conversion of cholesterol to bile acids and upregulation of hepatic LDL receptor expression. The net result is a reduction in the levels of circulating LDL-C [1,60,61]. Cholestyramine was studies in Lipid Research Clinics Coronary Primary Prevention Trial (LRC-CPPT). Cholestyramine reduced LDL-C by 20.3%. The reduction found in the treatment group was 12.6% higher than the reduction recorded in the placebo group. The cholestyramine group experienced a 19% reduction in the frequency of primary events, which include cardiovascular mortality or myocardial infarction [62]. Colestipol is not well studied. The efficacy of Colesevelam as a standalone treatment was evaluated in two clinical trials including patients with moderate primary hypercholesterolemia. After two weeks of treatment, the highest reduction in LDL-C relative to the initial level was 19% [63,64]. A meta-analysis utilizing mendelian randomization was conducted on 19 trials involving a total of 7021 study participants. The analysis revealed a decrease in LDL-C levels by 23.5 mg/dL and a tendency towards a lower risk of coronary artery disease (OR 0.81, 95% CI 0.70 to 1.02; p = 0.07) with the use of cholestyramine. Additionally, colesevelam was found to reduce LDL-C levels by 22.7 mg/dL (95% CI −28.3 to −17.2). There was no description of baseline LDL-C in these studies [65]. Constipation, bloating, and abdominal discomfort are common side effects of BAS therapy. Despite their efficacy, one should be mindful of gastrointestinal effects, in particular potential interference with vitamin absorption [66,67]. Due to modest impact on LDL-C and side effects, these medications are rarely used in the management of hyperlipidemia.

PCSK9 inhibitors

PCSK9 inhibitors are a novel group of medicines, recently approved for management of hyperlipidemia, particularly with those individuals in whom LDL target is not met despite maximally tolerated doses of statins, patients with FH, or those at high risk of cardiovascular events. PCSK9 is a protein produced by hepatocytes, it binds to LDL receptors on the surface of liver cells, promoting their degradation and reducing the ability of liver to remove LDLC from the bloodstream. Alirocumab and evolocumab are monoclonal antibodies that specifically attach to circulating PCSK9, preventing it from interacting with the LDL receptors. Inhibition of PCSK9 protein by these monoclonal antibodies allows the LDL receptors to remain on the hepatocyte surface increasing their availability to clear LDL-C from blood [68,69].

Clinical trials have shown that both alirocumab and evolocumab are very tolerable and effective in lowering LDL-C 60-70% when used as monotherapy or in combination with statins, that is, even greater reductions than statin therapy alone. In addition to LDL-C lowering, PCSK9 inhibitors have been shown to decrease the incidence of cardiovascular events, including myocardial infarction and strokes, in high-risk patients [70-72]. Significantly, the ACC/AHA recommendations state that PCSK9 inhibitors should be used in patients with ASCVD, a baseline LDL-C level of ≥190 mg/dL, and inadequate decrease in LDL-C (less than 50%) after statin treatment [73].

PCSK9 inhibitors are relatively safe and well tolerated medications with the adverse effects comparable to placebo or standard therapy. Long-term safety data is still being collected, particularly the potential for adverse effects on liver function, neurocognitive function, and immunogenicity (Table 3).

**Table 3:** Major PCSK9 inhibitor trials CHD: Coronary heart disease; CI: confidence interval; HR: hazard ratio; MI: myocardial Infarction; SPIRE: Studies of PCSK9 Inhibition and the Reduction of Vascular Events; PCSK9:  proprotein convertase subtilisin/kexin type 9

Study Name/ Publication Year	Sample Size	Inclusion Criteria	Study Type (Primary/Secondary Prevention)	PCSK9 Inhibitor Drug Name, Dose and Compared Against	Median Follow-Up	LDL Reduction	Primary End Point	Secondary End Point	HR and 95% CI
ODYSSEY OUTCOMES (2018) [74]	18,924	Patients with recent acute coronary syndrome	Secondary Prevention	Alirocumab 75 mg/150 mg vs. placebo	2.8 years	54.7% reduction	15% reduction in composite of CHD death, MI, ischemic stroke, or unstable angina requiring hospitalization	No significant difference in all-cause mortality	HR: 0.85 (95% CI: 0.78-0.93)
FOURIER (2017) [75]	27,564	Patients with atherosclerotic cardiovascular disease and LDL-C ≥70 mg/dL	Secondary Prevention	Evolocumab 140 mg every 2 weeks or 420 mg monthly vs. placebo	2.2 years	59% reduction	15% reduction in composite of cardiovascular death, MI, stroke, hospitalization for unstable angina, or coronary revascularization	20% reduction in MI, 27% reduction in stroke	HR: 0.85 (95% CI: 0.79-0.92)
SPIRE-1 (2017) [76]	16,817	Patients with hypercholesterolemia at low cardiovascular risk	Secondary Prevention	Bococizumab 150 mg every 2 weeks vs. placebo	7 months	54% reduction	No significant difference in primary end points	Not specified	HR: 0.99 (95% CI: 0.84-1.17)
SPIRE-2 (2017) [76]	10,621	Patients with hypercholesterolemia and established cardiovascular disease or at high risk	Secondary Prevention	Bococizumab 150 mg every 2 weeks vs. placebo	12 months	56% reduction	Significant reduction in primary end points: 179 events in the bococizumab group vs. 224 in the placebo group	Not specified	HR: 0.79 (95% CI: 0.65-0.97)
ODYSSEY LONG TERM (2015) [77]	2,341	Patients with heterozygous familial hypercholesterolemia or high-risk patients with hypercholesterolemia	Secondary Prevention	Alirocumab 150 mg every 2 weeks vs. placebo	78 weeks	62% reduction	Significant reduction in LDL-C levels: 62% at 24 weeks	Not specified	HR: 0.52 (95% CI: 0.31-0.90)
LAPLACE-2 (2014) [78]	1,896	Patients with hypercholesterolemia on statin therapy	Secondary Prevention	Evolocumab 140 mg every 2 weeks or 420 mg monthly vs. placebo	12 weeks	59-61% reduction	Significant reduction in LDL-C levels: 59-61% at 12 weeks	Not specified	HR: Not specified
ODYSSEY COMBO I (2014) [79]	316	Patients with hypercholesterolemia on statin therapy	Secondary Prevention	Alirocumab 75 mg/150 mg vs. placebo	52 weeks	50% reduction	Significant reduction in LDL-C levels: 50% at 24 weeks	Not specified	HR: Not specified
ODYSSEY COMBO II (2015) [80]	720	Patients with hypercholesterolemia on statin therapy	Secondary Prevention	Alirocumab 75 mg/150 mg vs. ezetimibe 10 mg	52 weeks	51% reduction	Significant reduction in LDL-C levels: 51% at 24 weeks	Not specified	HR: Not specified

Lomitapide

Lomitapide is an oral medication that inhibits the microsomal triglyceride transfer protein (MTP), a protein essential for assembly and secretion of apolipoprotein B containing lipoproteins in the liver and intestine. By inhibiting MTP, lomitapide prevents formation of VLDL in liver and chylomicrons and intestine. It has been licensed for use in combination with other therapies that lower lipid levels, primarily to manage severe hyperlipidemia, particularly in patients with HoFH [81]. Lomitapide reduces LDL-C and TG levels by 40% to 50% in patients with HoFH when used in conjunction with other lipid-lowering therapies by directly decreasing the formation of apo B-containing lipoproteins in the liver and gut [81,82]. Typical side effects include indigestion, stomach pain, feeling sick, loose stools, and vomiting [83]. Lomitapide treatment can interfere with the absorption of fat-soluble vitamins add essential fatty acids, thus fat-soluble vitamin supplementation may be required. Lomitapide is exclusively accessible through a limited Risk Evaluation and Mitigation Strategy (REMS) program due to the potential danger of treatment-induced hepatotoxicity.

Bempedoic acid

Bempedoic acid is a relatively new orally administered LDL-C-lowering agent, approved by the FDA in 2020, which works by inhibiting ATP-citrate lyase enzyme, specifically targeting the HMGCR enzyme at an earlier stage, leading to decreased cholesterol synthesis and increased clearance of LDL from the blood [84]. It is used for patients who are intolerant to statins or require additional LDL-C reduction despite maximally tolerated statin therapy. It can be used as a standalone treatment at a dose of 180 mg (sold as Nexletol in the United States) or in combination with ezetimibe at a dose of 10 mg (sold as Nexlizet in the United States) [85]. Possible indications for bempedoic acid, either alone or in combination with ezetimibe or PCSK9 inhibitors, involve assisting patients in achieving lower levels of LDL cholesterol than what can be accomplished with the highest tolerated dose of statins. Bempedoic acid at a daily dose of 180 mg effectively lowers LDL-C levels by 15% to 20%, whether used alone or in combination with statin therapy, and 50% reduction in LDL-C levels when used administered along ezetimibe 10 mg daily [86].

RNA interference (RNAi) therapies

Inclisiran

Inclisiran, also known as Leqvio and manufactured by Novartis, is a novel subcutaneously used lipid-lowering agent that utilizes RNA interference technology to reduce LDL-C levels. It is indicated for adults with heterozygous FH or established atherosclerotic cardiovascular disease who require additional lowering of LDL-C despite maximally tolerated statin therapy (Table 4). Inclisiran is a double stranded small interfering RNA (siRNA) molecule designed target and degrade the messenger RNA (mRNA) of proprotein convertase subtilisin/kexin type 9 (PCSK9) [87,88]. By silencing PCSK9 gene, it reduces production of PCSK9 protein. PCSK9 is involved in degradation of LDL receptors on hepatocytes. Lower levels of PCSK9 protein lead to increased availability and recycling of LDL receptors on hepatocytes, thus enhancing clearance of LDL-C from the bloodstream [88]. Inclisiran utilizes a siRNA-based approach to hinder PCSK9, which sets it apart from monoclonal antibodies. It is used in combination with statins and other lipid lowering therapies to achieve greater reduction in LDLC. Inclisiran effectively lowers LDL cholesterol levels by approximately 50% to 60% [89,90]. Inclisiran has extended the duration of effectiveness due to long-acting nature of the siRNA mechanism; it continues to lower both circulating PCSK9 and LDL-C levels for a period of 6 to 12 months following a single injection. Therefore, following the initial dose, the second dose is provided at three months, and then maintenance doses are administered every six months. A meta-analysis demonstrated that inclisiran significantly decreased the likelihood of major ASCVD events, with a risk ratio of 0.76 (95% confidence interval, 0.61-0.92, p < 0.01) [87]. Except for a higher occurrence of minor injection site responses, there were no discernible differences in adverse effects between the groups [87,89].

**Table 4:** Major inclisiran trials LDL-C: Low-density lipoprotein cholesterol

Study Name/ Publication Year	Sample Size	Inclusion Criteria	Study Type (Primary/Secondary Prevention)	Dose of Inclisiran and Compared Against	Median Follow-Up	LDL Reduction	Primary End Point	Secondary End Point	HR and 95% CI
ORION-1 (2017) [91]	501	Patients with elevated LDL-C on maximally tolerated statin therapy or with statin intolerance	Secondary Prevention	Inclisiran 200 mg, 300 mg, or 500 mg vs. placebo	6 months	Up to 41.9% reduction	Significant LDL-C reduction with Inclisiran	Not specified	Not specified
ORION-10 (2020) [92]	1,561	Patients with atherosclerotic cardiovascular disease and elevated LDL-C	Secondary Prevention	Inclisiran 300 mg vs. placebo	18 months	Up to 52% reduction	15% reduction in primary end point events: 7.8% in Inclisiran group vs. 10.3% in placebo group	Not specified	HR: 0.85 (95% CI: 0.72-1.00)
ORION-11 (2020) [92]	1,617	Patients with atherosclerotic cardiovascular disease or risk equivalents and elevated LDL-C	Secondary Prevention	Inclisiran 300 mg vs. placebo	18 months	Up to 49% reduction	15% reduction in primary end point events: 7.7% in Inclisiran group vs. 10.3% in placebo group	Not specified	HR: 0.85 (95% CI: 0.73-1.00)
ORION-4 [93]	16,124	Patients with atherosclerotic cardiovascular disease	Secondary Prevention	Inclisiran 300 mg vs. placebo	5 years		Ongoing trial with expected LDL-C reduction	Ongoing trial with expected cardiovascular outcomes	Ongoing trial

ANGPLT3 inhibitors

Evinacumab, also known as Evkeeza and manufactured by Regeneron, is a promising new type of lipid-lowering agent approved as adjunct to other LDL-C lowering therapies for adults and pediatric patients aged five years and older with homozygous familial hypercholesterolemia (HoFH). It works by inhibiting Angiopoietin-Like 3 (ANGPLT3) protein. ANGPLT3 inhibits LPL and endothelial lipase (EL) enzymes, which are critical for breakdown of triglycerides and phospholipids [94,95]. By enabling these enzymes, ANGPLT3 increases levels of triglycerides and lipids in the bloodstream. Inhibition of ANGPLT3 by evinacumab leads to increased activity of LPL and EL resulting in decreased levels of triglycerides, LDL-C, and other lipoproteins. When used as an add on therapy to other lipid-lowering agents including statins, ezetimibe, lomitapide, PCSK9 inhibitors, and apheresis, evinacumab caused approximately 50% reduction in LDL-C from baseline [96,97].

Novel therapies under investigation

Current treatment options for hyperlipidemia largely consist of statins, fibrates, ezetimibe, and PCSK9 inhibitors. Inclisiran is a novel agent and is a recent addition to management of hyperlipidemia. However, there is a continuous need for new therapeutic strategies, especially for patients who are intolerant to existing medications or do not achieve desired lipid levels. Several novel therapies for management of hyperlipidemia are under investigation and are at various stages of development to the fulfill the unmet needs of lipid management. Some of such newer agents are discussed as below.

ApoC-III inhibitors

Gemcaebene

Gemcabene is an experimental orally administered lipid-lowering drug with potential use as an adjunct to statins in patients with FH, monotherapy or in combination with other lipid-lowering agents in patients with hypertriglyceridemia, and in non-alcoholic fatty liver disease due to its lipid-lowering and anti-inflammatory effects [98]. Gemcaebene is small molecule that has a symmetrical molecular structure consisting of dicarboxylic acid and two terminal gem dimethyl carboxylate moieties. Gemcabene inhibits hepatic lipid synthesis of Apolipoprotein c-III (ApoC-III), and lower levels of ApoC-III enhance the breakdown and removal of lipoproteins from blood. It also activates peroxisome proliferator-activated receptor-alpha (PPAR-α), leading to increased fatty acid oxidation and reduced triglyceride levels and liver. It has also been shown to reduce the levels of pro-inflammatory cytokines, contributing to its potential benefits in inflammatory conditions like NAFLD [78,98,99]. The daily doses of 300 and 900 mg have shown a reduction in LDL-C levels by 23% and 28% respectively, when used in conjunction with statin therapy. Gemcabene effectively decreased LDL-C levels by around 30% in individuals with biallelic hypercholesterolemia (HoFH) [100]. While clinical trials have demonstrated its efficacy and safety in short term, further studies are needed to confirm its long-term benefits and optimal use.

DGAT2 inhibitors

Diacylglycerol O-acyltransferase 2 (DGAT2) inhibitors are promising ones aimed at the treatment of metabolic disorders, particularly non-alcoholic steatohepatitis, type 2 diabetes, and hyperlipidemia. DGAT2 is an enzyme involved in triglyceride synthesis, making it a potential target for reducing lipid accumulation in the liver and other tissues [101-103]. IONIS-DGAT2Rx (ISIS 703802), ARO-DGAT2, PF-05221304, and PF-06424439 are still largely in the investigational stage, these drugs aim to reduce triglyceride synthesis and accumulation, thereby improving metabolic health, and are the drugs under investigation [101,102,104].

ACAT2 inhibitors

Acyl-CoA: cholesterol acyltransferase 2 (ACAT2) is one of the two acyl-coenzyme A: cholesterol acyltransferase (ACAT), also known as sterol O-acyltransferase (SOAT), primarily expressed in the liver and intestine, where it catalyzes formation of cholesterol esters, a process crucial for formation and secretion of VLDL in the liver, and absorption of dietary cholesterol in the intestine. ACAT2 inhibitors are an emerging class of investigational drugs, aim to reduce cholesterol esterification and absorption, thereby lowering plasma cholesterol levels and reducing the risk of atherosclerosis and hypercholesterolemia [105,106]. CP-113, 818, K-604, and pyripyropene A are studied on animal models and have shown reduction in absorption of dietary cholesterol, decreased plasma cholesterol level, and reduced formation of atherosclerotic plaque formation [107-109]. Avasimibe, and ACAT2, did show potential in reducing plaque formation, but is discontinued due to adverse effects and limited efficacy [106]. Other drug that did not advance due to similar issues include Pctimibe, Sandoz 58-035, and F12511.

LPL gene therapy medication

LPL gene therapy is a novel approach to treat patients LPL gene deficiency or mutation by incorporating LPL gene copies. Lipoprotein lipase is critical for the hydrolysis of triglycerides in chylomicrons and VLDL into free fatty acids and glycerol, which are then taken up by tissues for energy production or storage. LPL deficiency is a rare but severe condition, which impair this process, leading to extremely high triglyceride levels and recurrent pancreatitis [110,111]. Glybera is gene therapy, which delivers LPL gene using adeno-associated viruses vector. It was approved in 2012 for the treatment of familial LPL deficiency. Clinical trials demonstrated significant reductions in TG levels and a decrease in the frequency of pancreatitis episodes. However, due to high costs and limited market demand, Glybera was withdrawn from the market in 2017 [111].

## Conclusions

Hyperlipidemia is a multifactorial condition and remains a significant and modifiable risk factor for cardiovascular diseases, necessitating a comprehensive understanding of its pathophysiology and management. While genetics play a significant role, lifestyle factors such as diet, exercise, and smoking also contribute significantly to hyperlipidemia. Prevention and treatment of hyperlipidemia are crucial to reduce cardiovascular mortality and morbidity. Lifestyle modification, drug treatment, and emerging therapies are critical for achieving optimal lipid control and prevention and progression of ASCVD. Traditional treatments like statins and fibrates have proven efficacy, while newer agents such as PCSK9 inhibitors, bempedoic acid, and RNA interference therapies like inclisiran expand the therapeutic arsenal. Investigational drugs and emerging therapies hold promise for patients with refractory hyperlipidemia, potentially transforming future management strategies. Advances in genetic research are improving the understanding of its mechanisms, leading to better diagnostic, preventive, and therapeutic strategies. Genetic testing and personalized medicine are becoming increasingly important in the management of hyperlipidemia.
